# Cytokines and Other Mediators in Alopecia Areata

**DOI:** 10.1155/2010/928030

**Published:** 2010-03-11

**Authors:** Stamatis Gregoriou, Dafni Papafragkaki, George Kontochristopoulos, Eustathios Rallis, Dimitrios Kalogeromitros, Dimitris Rigopoulos

**Affiliations:** Andreas Sygros Hospital, University of Athens Medical School, 5 Ionos Dragoumi Street, 16121 Athens, Greece

## Abstract

Alopecia areata, a disease of the hair follicles with multifactorial etiology and a strong component of autoimmune origin, has been extensively studied as far as the role of several cytokines is concerned. So far, IFN-*γ*, interleukins, TNF-*α*, are cytokines that are well known to play a major role in the pathogenesis of the disease, while several studies have shown that many more pathways exist. Among them, MIG, IP-10, BAFF, HLA antigens, MIG, as well as stress hormones are implicated in disease onset and activity. Within the scope of this paper, the authors attempt to shed light upon the complexity of alopecia areata underlying mechanisms and indicate pathways that may suggest future treatments.

## 1. Introduction

Alopecia areata is a disease of the anagen stage hair follicles, considered to be autoimmune in origin. Prevalence in the general population is between 1% and 2% [1] and it can occur at any age. Alopecia areata is characterized by either patchy hair loss or more generalized alopecia that results in complete loss of scalp hair (alopecia totalis). The presence of atopy and the onset in prepubertal children indicates poor prognosis [2]. The histopathologic features of the disease consist of perifollicular lymphocytic infiltrates around anagen hair follicles, consisting of both CD4+ and intrafollicular infiltrates of CD8+ cells. Several studies have shown that within the cascade of pathogenesis of alopecia areata, cytokines and other molecules that coordinate cyclical hair growth play a crucial role. In this paper, we attempt to review the role of some of the most important cytokines, along with other mediators in alopecia areata, and indicate pathway routes that may be target therapies in the future.

## 2. IFN-*γ*

IFN-*γ* is the main cytokine known to be aberrantly expressed in alopecia areata through a CD4+ Th1 mediated response. IFN-*γ* is produced by perifollicular or follicular antigen presenting cells and among several actions it also deprives dermal papilla cells of their ability to maintain anagen hair growth, as shown in human studies [3]. It has been shown that serum levels of IFN-*γ* are significantly higher in patients with alopecia totalis or alopecia universalis compared to controls, but no significant difference has been found in levels of IFN-*γ* between patients with localized alopecia areata and those with more extensive forms [4]. Additionally, experiments in human alopecia areata by Deeths et al. show that antigen specific T-cells from patients with extensive disease appear to have some intrinsic defect towards production of IFN-*γ*, possibly suggesting a state of partial tolerance in the skin of these patients [5]. Despite the above, the elevated serum levels of IFN-*γ* in alopecia areata patients may reflect the state of inflammation, especially in the extensive forms of the disease, and the measurement of serum IFN-*γ* may be useful in discriminating those who are likely to develop alopecia universalis from the remaining local disease, or as a prognostic indicator. It is suggested that future studies may be able to evaluate the evolution of IFN-*γ* levels in patients with spontaneous regression or progressive extension of the disease [4]. 

MIG (monokine induced by IFN-*γ*) is a cytokine that is elevated in human alopecia areata and its level correlates with disease activity, increasing in expanding lesions and vice versa, making it a useful marker of monitoring of the disease status and response to treatment [6]. MIG mRNA is mostly found in mononuclear cells in the peri- and intrabulbar infiltrate and also in the follicular papilla. Also, another chemokine leading to recruitment of mononuclear cells is IP-10 (interferon inducible protein-10), which is also induced by IFN-*γ*. Experiment in IP-10 is much less expressed compared to MIG but accounts for the persistence of Th1 response in alopecia areata, perpetuating the recruitment of lymphocytes [7].

## 3. Interleukins

Studies have shown that IL-1 is a very potent inducer of hair loss and a significant human hair growth inhibitor in vitro. Hoffman et al. have demonstrated that during induced murine hair cycle, IL-1*α* and IL-1*β* increase profoundly with the onset of spontaneous catagen phase, while they peak during telogen phase and are associated with increased expression of the signal transducing type I IL-1 receptor [8]. Additionally, according to studies by Groves et al., transgenic mice overexpressing IL-1a in the epidermis have patchy hair loss resembling alopecia areata [9]. 

In human scalp areas affected by alopecia areata, an excessive expression of IL-1*β* is detected particularly at the early stages of the disease, while susceptibility to the disease and severity are determined by polymorphisms of the IL-1-receptor antagonist and IL-1a. In terms of clinical severity, a more progressive expression of the disease is encountered in patients, who, due to gene polymorphisms, have insufficient amounts of IL-1 receptor antagonist, the natural antagonist of IL-1 [10], while an increased frequency of the allele 2 of the IL-1 receptor antagonist gene was found in patients with extensive AA hair loss [11]. Experiments in cultured hair follicles by Philpott et al. showed that the effects of IL1-*α* and IL1-*β* on them may be blocked by addition of the IL-1 receptor antagonist [12]. Also, IL-1 gene polymorphisms may be responsible for exaggerated release of IL-1, leading to rapid and more progressive disease [13]. Galbraith et al. showed that patients with severe forms of alopecia areata have an increased frequency of the IL-1*β* 1,2 genotype [14], with allele 2 of the IL-1*β* +3953 polymorphism exhibiting a strong association with increased production of IL-1*β* [15]. In the same study it was found that IL-1*β* loci along with loci of immunoglobulin *κ* light chain act cooperatively to significantly increase susceptibility to the disease [14]. 

Serum levels of IL-1*α* and IL-4 are significantly elevated in patients with localized alopecia areata, while IL-2 and IFN-*γ* are mainly elevated in extensive disease states, possibly implying that the progression to the extensive form may be mediated by Th1 cytokines [16]. It is also considered that a disequilibrium in the production of cytokines, with a relative excess of proinflammatory and Th1 types, versus antiinflammatory cytokines, such as IL-4 and IL-10 may be involved in the persistence of alopecia areata lesions, as shown in human scalp biopsies [17]. Finally, in agreement with above, it has been shown that steady-state levels of IL-10 mRNA increase after successful DCP treatment, making IL-10 an important inhibitor of Th1 cytokine production [18]. 

## 4. TNF-*α*

TNF-*α* is well known to play a major role in the pathogenesis of alopecia areata. TNF-*α* is synthesized in epidermal keratinocytes along with several other cytokines [19] and is known to be a very potent inhibitor of proliferation [20]. In vitro studies have shown that TNF-*α*, along with IL-1*α* and IL1-*β*, causes vacuolation of matrix cells within the follicle bulb and a decrease in the size of the matrix, as well as disorganization of follicular melanocytes and abnormal differentiation and keratinization of the precortical cells and the inner root sheath [12]. TNF-*α* levels in the skin correlate positively with plasma ACTH levels and cutaneous ACTH receptor expression levels under repeated stress in humans [21], possibly suggesting a pathophysiologic mechanism lying behind the well-known role of stressors in alopecia areata. 

The sera of patients with alopecia areata and in particular, the subgroup of patients with multiple lesions, have been found to contain extremely high levels of BAFF, namely, B cell activating factor that belongs to the TNF family, produced by myeloid lineage cells [22]. It is considered that the production of BAFF is stimulated by IFN-*γ* that is well known to be increased in alopecia areata patients, as mentioned above [23]. Experiments in mice have shown that BAFF may also activate T-cells and thus promote Th1 response, leading to the production of IFN-*γ* and perpetuation of disease activity [24]. 

## 5. Major Histocompatibility Complex (MHC) and Fas-Antigen

The hair follicle is a frequent target of immune-mediated tissue injury, leading to development of alopecia areata. Under normal conditions, the hair follicle is considered an area of relative immune privilege during the anagen stage of hair growth. Thus, autoantigens are not recognized by CD8+ T-cells, allowing normal hair growth. MHC class I antigens demonstrate very low expression during this phase, something which is mediated by locally produced cytokines, such as TGF-b1, ACTH, a-MSH, and IGF-1, all of which serve as very potent immunosuppressants, produced by anagen hair bulbs [25]. Also, anagen hair bulbs show very few antigen-presenting cells that appear to be functionally impaired since they do not express MHC class II antigens [26]. 

Alopecia areata lesions can be induced in mice by the transfer of MHC class I restricted CD8+ T-cells alone, while anagen hair follicle antigens are essential for T-cell stimulation that ends up in the development of the disease [27]. CD8+ cells are of crucial importance in AA, by interacting with MHC-I restricted autoantigens and inducing cytolysis of target cells [10]. Several triggers, such as emotional factors, skin microtrauma, or infectious agents aided by a possible underlying immune predisposition, lead to intrafollicular rise in IFN-*γ*, that causes MHC class Ia upregulation in the proximal hair follicle epithelium [25]. Also, HLA class II antigens and ICAM-1, both implicated in lymphocyte trafficking and antigen presentation, increase their expression in response to stimuli by IFN-*γ* and TNF-*α* in cultured hair follicles [28]. 

Fas-antigen belongs to a nerve growth/TNF receptor superfamily and it is a membrane protein involved in apoptosis. It is considered that the abnormal expression of Fas-antigen on hair follicles keratinocytes may play an important role in alopecia areata [17]. 

## 6. The Role of Mononuclear Cells

The regulatory role of peripheral blood mononuclear cells (PBMCs) has been clearly elucidated through the work of Zöller et al. It has been found that active alopecia areata patients' PBMCs display increased resistance towards apoptosis. This is considered to be sustained by downregulation of CD95L+ and increase in CD44v7+, both known to be associated with antiapoptotic gene expression. PBMC from patients with progressive alopecia areata reveal a higher percentage of CD4+ CD25+ CD154+ T-cells that suppress the proliferative activity of CD8+ PBMC to a much lesser extent when compared with CD4+ CD25+ PBMC of health donors or patients with stable or regressive alopecia areata. In particular, it is considered that the population of CD4+ CD25+ CD154+ PBMC that express CD44v7+ is very resistant to apoptosis and characterizes active alopecia areata [29]. 

## 7. Macrophage Migration Inhibitory Factor (MIF)

MIF is a cytokine produced by lymphocytes and peripheral blood mononuclear cell that may play a key role in the pathogenesis of extensive alopecia areata. MIF was the first lymphokine reported to prevent the random migration of macrophages [30]. MIF levels are significantly elevated in alopecia areata patients. This molecule stimulates the production of IL-1 and TNF-a by macrophages, while the latter exert positive feedback effect on MIF. The cycle endpoint is an on-going inhibition of hair growth [31]. Polymorphisms within the MIF-173C allele confer an increased risk of early onset extensive form of alopecia areata at ages below 20, as shown in the study of Shimizu et al. [32]. Given the fact that antibodies against MIF have shown encouraging results in the treatment of murine hepatitis [33], MIF production control may show promise for effective treatment modalities in extensive alopecia areata patients. 

## 8. Alopecia Areata and Stress Hormones

In alopecia areata, significant interactions have been found between the neuroendocrine and the immune system. Experimental studies in mice with alopecia areata showed altered hypothalamic-pituitary-adrenal (HPA) axis activity, possibly secondary to altered immune response. In particular, a marked increase in the HPA axis tone, and activity was noted, both in central and peripheral levels in the skin and lymph nodes, as well as a significantly blunted ACTH response to acute stress and repeated restraint stress [34]. Other studies showed increased ACTH and *α*-MSH levels in alopecia areata patients, suggesting the presence of an active neurogenic system and local HPA axis activity, with positive correlations with TNF-*α* level, as mentioned above. Additionally, hypothalamic arginine vasopressin mRNA and pituitary proopiomelanocortin mRNA were found to be elevated under acute and repeated stress conditions, also suggesting HPA axis hyperactivity [34]. Furthermore, the decreased hypothalamic and hippocampal expression of estrogen receptor-*β* under basal and repeated stress, and the increased expression under acute stress possibly indicate a disrupted hypothalamic pituitary gonadal axis in alopecia areata disease states [21]. The above may well provide a strong evidence for the role of stress hormones in modulating the inflammatory response in this category of patients.

## 9. Diphenylcypropenone (DCP)

The role of cytokines in the pathogenesis of alopecia areata has been elucidated through the use of DCP, a contact sensitizer and basic treatment modality for this disease. It has been suggested that treatment of alopecia areata lesions with a contact sensitizer influences leukocyte migration, causing increase in monocytes and decrease in dendritic cell migration towards draining nodes [35]. It is suggested that the beneficial effect of DCP is mediated by locally secreted cytokines during the contact allergy [36]. Happle et al. have proposed the Renbok phenomenon as the opposite of Koebner phenomenon, to describe the response of alopecia areata diseased skin to contact sensitizers [37]. 

In DCP treated patients, a perifollicular infiltrate mainly composed of CD8+ and CD1a cells is noted, as well as increased expression of CD44 and CD49d. The increase in CD1a + dendritic cells indicates hampering of the emigration of antigen presenting cells by DCP treatment, while both CD44 and CD49d play major role in leukocyte extravasation [38]. After successful DCP treatment the ratio of CD4 : CD8 decreases from 4 : 1 to 1 : 1 in the perifollicular infiltrate and the expression of IFN-*γ* decreases along with decrease in IL-1*β* and increases in IL-2, IL-8, IL-10, and TNF-a [6]. 

DCP use is associated with an elevated number of infiltrating leukocytes in the bulbar and suprabulbar area of the hair follicle, apparently newly recruited leukocytes that act against autonomously functioning CD4+ and CD8+ cells. The above process is mediated by several cytokines, as mentioned above, implicating that pathogenesis and recovery form alopecia areata follow a well-programmed pathway.

## 10. Discussion

A concrete hypothesis on the pathogenetic mechanisms of alopecia areata, as it becomes evident through the role of the above mentioned cytokines and their pathways, is difficult to be formed. It has been suggested that a T-cell mediated autoimmune process is triggered by endogenous or exogenous stimuli and maintained by the interaction of several molecules. Probably, in genetically predisposed individuals, under the effect of stress hormones, the hair follicle enters a cycle of autoimmune inhibition of its growth. Although alopecia areata is considered to have immunologic origin, neither autoimmune disorders nor autoantibodies are more common in these patients. The pathogenetic mechanisms so far known indicate a rather complex process that sustains an inflammatory reaction, that is, a vicious cycle which leads to hair loss. The keratinocytes release cytokines that activate endothelial cells, which in turn attract T-cells and macrophages that release more cytokines. 

Despite the complicated pathogenesis of alopecia areata, several studies over the last years have significantly attempted and managed to throw light into the mechanisms of origin and evolution of this entity. So far well-known cytokines, such as TNF-*α*, interleukins, and IFN-*γ*, have been extensively studied along with many more, and possible therapeutic targets have been identified. Interestingly, antiTNF-*α* drugs have not proven to be of use in alopecia areata. According to the recent observational studies, alopecia areata lesions have developed in patients receiving such treatments [39] and recurrence of disease has occurred while receiving antiTNF-*α* drugs [40]. Similarly, in other dermatologic diseases, such as psoriasis, it has been shown that antiTNF-*α* may have a paradoxical adverse event, leading to the development of psoriatic skin lesions in predisposed individuals [41]. A suggested explanation of this failure might be that TNF-*α* is just one of the ever expanding list of cytokines involved in the pathogenesis of alopecia areata. Additionally, in other immune-mediated inflammatory diseases, such as psoriasis, where anti TNF-a agents constitute a within label treatment, different antiTNF-*α* agents display variable efficacy in different patients. An antiTNF-*α* drug may also fail to maintain the initial good response over time, possibly due to the development of neutralizing antibodies. Definitely, the role of TNF-*α* in the pathogenesis of alopecia areata as well as other autoimmune diseases has to be further investigated as far as treatment with antiTNF-*α* agents is considered. 

Nevertheless, inability to target therapeutically a single cytokine suggests that alopecia areata is either so multifactorial in its pathogenesis, or, we only see the tip of the iceberg. Despite that, the plethora of implicated mechanisms that potentially give onset to and maintain or increase the activity of this disease may show promise for future treatment modalities that will make a real difference in the lives of these patients.
